# The relationship between paediatric foot posture and body mass index: do heavier children really have flatter feet?

**DOI:** 10.1186/s13047-015-0101-x

**Published:** 2015-08-27

**Authors:** Angela Margaret Evans, Leila Karimi

**Affiliations:** Department of Podiatry, Lower Extremity and Gait Studies (LEGS) Research Program, La Trobe University, Bundoora, Melbourne, Australia; School of Public Health and Human Biosciences, La Trobe University, Bundoora, Melbourne, Australia

**Keywords:** Flatfeet, Children, Foot posture, Body mass index, BMI, Paediatric, Weight

## Abstract

**Background:**

Several studies have found positive correlation between flatfeet and increased body mass in children. One study, utilizing a differing method of foot posture assessment, found the inverse. The purpose of this study was to further explore the relationship between children’s foot posture and body mass, utilizing the foot posture index in a large study population, as opposed to the footprint based measures of most previous studies.

**Methods:**

Data for both foot posture index (FPI) and body mass index (BMI) for healthy children were acquired from five previous studies. The amalgamated dataset comprised observations for both BMI and FPI-6 in 728 children aged from three to 15 years. Three FPI-6 scores levels defined the range of flatfeet detected: FPI-6 ≥ +6; FPI-6 ≥ +8; FPI-6 ≥ +10. BMI cut-points were used to define overweight for each age group.

**Results:**

In the study population of 728 children, flatfeet (FPI ≥ +6) were found in 290 (40 %) cases and non-flatfeet in 438 (60 %) cases. FPI ≥ +8 yielded flatfeet in 142 (20 %) cases and FPI ≥ +10 yielded flatfeet in 41 (5 %) cases. Whilst 272 (37 %) children were overweight, only 74 (10.1 %) of the overweight children had flatfeet (FPI ≥ +6), which diminished to 36 (4.9 %) at FPI ≥ +8, and 9 (1.2 %) at FPI ≥ +10.

Significant and moderate correlation was found between BMI and age (*r* = 0.384, *p* < 0.01). Very weak, but significant, correlation was found between BMI and FPI (*r* = −0.077, *p* < 0.05). Significant mean differences between gender and BMI were found (*t*-test = 2.56, *p* < 0.05). There was strong correlation between FPI scores on left and right sides (*r* = 0.899, *p* < 0.01).

**Conclusions:**

This study found no association between increased body mass and flatfeet in children, a finding in contrast to that repeatedly concluded by many previous studies. Whilst properties of the FPI and BMI are limiting, these findings question the concern about children’s increased body mass as a specific influence on (flatter) foot posture, and also the validity of footprint versus anatomically based foot posture measures.

## Background

The prevailing opinion, that heavier children have flatter feet, has been espoused and supported by the findings of several studies [1–8], and may seem to be an intuitive observation. Recently however, this premise has been queried, and subsequently it has been postulated that the method of foot posture assessment may be responsible for this repeated finding [5].

In the last decade especially, several studies have investigated the relationship between paediatric foot posture (viz. paediatric flatfoot) and anthropometry. Whilst many different measures of foot morphology have been used, all but one study included a version of footprints (printed, scanned, digitized) as a proxy measure for foot morphology, as is detailed in Table 1.

**Table 1:** Summary of the studies investigating the relationship between children’s foot posture and body mass

Date	Author, country	Ages (years)	Sample (N)	Methods of foot posture assessment	Flat feet related to body mass
2001	Dowling, Australia	8 - 9	26	Footprints, pressure mat	yes
2006	Pfeiffer, USA	3 - 6	835	Scanner, rearfoot angle	yes
2006	Mickle, Australia	4 - 5	38	Footprints, US fat pad measures	yes
2007	Morrison, UK	9 - 12	200	Foot length/width, navicular height	yes
2008	Mauch, Germany	2 - 14	2887	Scanner	yes
2009	Villarroya, Spain	9 – 16.5	58	Footprints, x-rays	yes
2009	Chen, Taiwan	5 - 13	1024	Footprints, 3D scan	yes
2010	Chang, Taiwan	7 - 12	2083	footprints	yes
2011	Evans, Australia	7 - 10	140	FPI-6	no
2013	Wozniacka, Poland	3 - 13	1115	Scanner	yes
2013	Jimemez-Ormeno, Spain	6 - 12	1032	3D digitizer	yes

A previous investigation found that both overweight and obesity were associated with flat foot posture in 835 children aged three to six years, where flat foot was found in 42 % of normal weight children, 51 % of overweight children, and 62 % of obese children [1]. Similarly, two Taiwanese studies in 1024 children aged five to 13 years [3], and 2083 children, aged between seven and 12 years of age found significant increase in the prevalence of flatfoot in overweight and obese children [4].

In Spain, a small study compared children’s footprint measures and foot x-rays with body mass categorized as normal-weight or obese in 58 children aged nine to 16 years [2]. This study found that the obese children had significantly lower arched feet as determined by footprint measures, and supported by x-ray findings. Likewise, a German study, using a scanner to investigate the influence of body mass on the development of a child’ s foot in 2887 children aged two to 14 years [6], and a Polish study which assessed foot posture and body mass in 1115 children, aged between three and 13 years [8], found positive correlation between arch height and increased body mass, with stronger correlation observed in girls.

Similar findings have been found in previous studies conducted on overweight and obese Australian children [9, 10].

Whilst not within the bounds of age-defined childhood, a recent and notable investigation in 17 year old adolescents assessed the association between body mass, gender and flatfoot [11]. This study of recruits for mandatory military service, included 825,964 adolescents (467,412 males; 358,552 females). Foot type was assessed by clinical observation of arch height and heel valgus (assessed with the subjects in static stance, and repeated with subjects standing on tip-toes). According to the visualized arch height or depression, subjects were categorized as having either mild or severe flatfoot. For males, mild flatfoot was found in 12.4 %, and severe flatfoot in 3.8 %, with females found to have mild flatfoot in 9.3 %, and severe flatfoot in 2.4 %. Increased BMI was significantly associated with flatfoot in both males (overweight: odds ratio [OR] 1.385, obese: OR 1.765, and females (overweight: OR 1.408; obese: OR 1.549). Interestingly, increased height was associated with decreased flatfoot findings in both males (OR 0.782) and females (OR 0.730) [11].

In contrast to all of these investigations which utilized a range of foot morphology measures (Table 1), our previous study of 140 school children found no direct relationship between increased body mass and flatter feet in children when utilizing the foot posture index (FPI-6) [5], creating the impetus for closer scrutiny and further investigation of this apparent anomaly.

Hence, the aim of this study was to examine the relationship between increased body mass as depicted by the body mass index (BMI), and foot posture using the FPI-6, in a larger sample of children.

## Methods

### Data acquisition

Data was acquired from multiple sources where both BMI and FPI-6 had been assessed in healthy children. Each dataset came from authors who had previously gained ethics committee approval for each individual dataset. All data was anonymised and connected with cited publications.

Two datasets were acquired from the author’s previous works investigating the reliability of clinical assessment measures (*n* = 170) [5, 12], and further datasets were acquired from other authors in the UK investigating foot posture in young children (*n* = 225) [13], and Australia investigating Sever’s disease (234 controls, 67 subjects) (*n* = 303 [14], and the control group from an idiopathic toe-walking study (*n* = 30), [15] to realize an amalgamated dataset of 728 observations for both BMI and FPI-6 in children aged from three to 15 years.

### Statistical analysis

Data were entered and all analyses were performed using constructed data sets in Microsoft Excel 2000 (Microsoft Inc, Redmond, Washington) and SPSS version 22 (SPSS Inc, Chicago, Illinois) software packages. Testing for normality using a Kolmogorov–Smirnov test, found normal distribution of data, directing parametric statistics for analysis. Descriptive statistics (mean, standard deviation, minimum, maximum, frequencies) were used to examine the basic anthropometrical characteristics of the study populations. Relationships between variables (notably BMI, FPI-6, and age) were analysed using the parametric correlation statistic of Pearson’s *r*. Student’s *t*-test was conducted to detect any significant differences between males and females and their level of BMI. An analysis of the correlations between BMI z-score with FPI was made, given that age as a potential confounding factor on the relationship between BMI and FPI.

The cut-off values for BMI per age group, established by the international obesity task force (IOTF), were used to determine normal versus overweight/obesity for each age/year group [16].

With reference to the available normative data [17], three FPI-6 scores levels were used to ‘define and explore’ the range of flatfeet detected: FPI-6 ≥ +6; FPI-6 ≥ +8; FPI-6 ≥ +10.

## Results

The mean age of the study population of 728 children was 9.07 years (SD 2.38), ranging from three years to 15 years. The mean BMI was 18.21 kg/m^2^ (SD 3.48), ranging from 10.57 kg/m^2^to 37.94 kg/m^2^. The mean FPI-6 score was 4.64 (SD 3.08) and 4.95 (3.31) for left and right feet respectively, ranging from −4 to +12 (left) and −3 to +12 (right). Specific case gender data was only available for *n* = 588 (325 male: 263 female). The total study population gender distribution was 375 male, 353 female.

The three FPI score levels (FPI-6 ≥ +6; FPI-6 ≥ +8; FPI-6 ≥ +10) respectively, yielded flatfeet in 290/142/41 children, non-flatfeet in 438/585/687 children. The mean BMI for the flatfeet groupings were: FPI +6 (*n* = 290) 18.10 kg/m^2^ (SD 3.65), minimum 10.57 kg/m^2^to maximum 30.54 kg/m^2^ (range 19.97 kg/m^2^); FPI +8 (*n* = 142) 17.73 kg/m^2^ (SD 3.56), minimum 10.57 kg/m^2^to maximum 28.84 kg/m^2^ (range 18.27 kg/m^2^); FPI +10 (*n* = 41) 17.39 kg/m^2^ (SD 3.09), minimum 12.76 kg/m^2^to maximum 25.11 kg/m^2^ (range 12.35 kg/m^2^).

In the non-flatfeet groups (those whose foot posture scored below the FPI cut-off levels) the mean BMI was: FPI +6 (*n* = 438) 18.38 kg/m^2^ (SD 3.49), minimum 11.70 kg/m^2^ to maximum 37.94 kg/m^2^ (range 26.24 kg/m^2^); FPI +8 (*n* = 585) 18.30 kg/m^2^ (SD 3.51), minimum 10.57 kg/m^2^ to maximum 37.94 kg/m^2^ (range 27.37 kg/m^2^); FPI +10 (*n* = 687) 18.25 kg/m^2^ (SD 3.49), minimum 10.57 kg/m^2^ to maximum 37.94 kg/m^2^ (range 27.37 kg/m^2^).

**Fig. 1:**
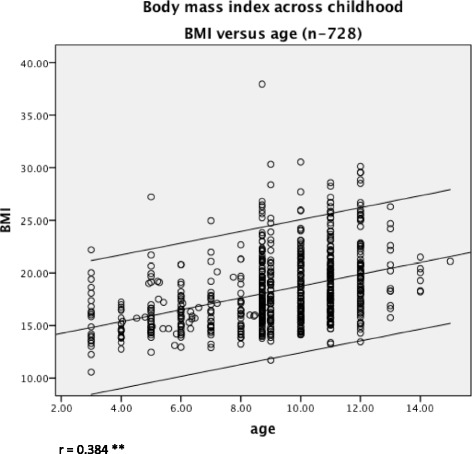
The relationship between children’s BMI and age. There was moderate relationship between increasing children’s BMI with increasing age, as indicated by the line of best fit and 95 % confidence intervals

**Fig. 2:**
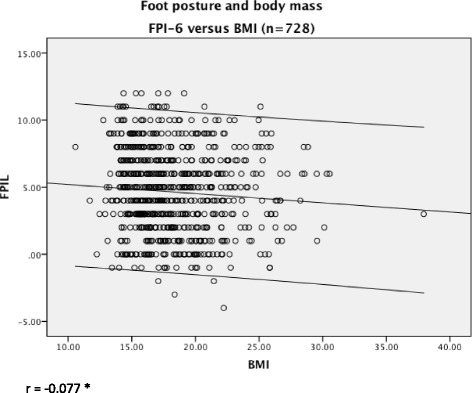
The relationship between children’s foot posture and BMI. The relationship between foot posture and body mass, as indicated by the line of best fit and 95 % confidence intervals, was weak and inverse, with correlation showing almost no relationship between body mass and foot posture

**Fig. 3:**
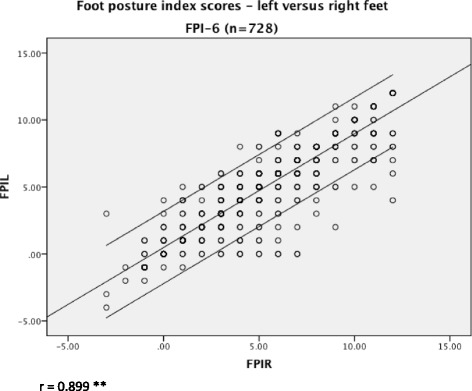
The relationship between children’s left and right foot postures. A strong correlation was found between left and right posture, meaning that the FPI-6 data for either foot was applicable for analyses

Significant and moderate correlation was found between BMI and age (*r* = 0.384, *p* < 0.01) (Fig. 1). The correlation between BMI and FPI, whilst also statistically significant, was very weak (*r* = −0.077, *p* < 0.05), as illustrated in Fig. 2. There was strong correlation between FPI scores on left and right sides (*r* = 0.899, *p* < 0.01), from which the left side was arbitrarily used for subsequent analyses (Fig. 3). Using *t*-test the relationship between BMI and gender was further investigated. The results showed significant gender differences (t = 2.56, *p* = 0.01) indicating the higher BMI in males (mean 18.53, SD = 3.71) compared to females (mean 17.79, SD = 3.20). Significant correlations were found between age and BMI (z score) (*r* =0.384, *p* = 0.01), and also between BMI (z score) and FPI (*r* = 0.078, *p* = 0.05).

Table 2 collates the study population of 728 children by age group, and flatfoot presence/range. From this, the children with flatfeet were calculated at each of the three selected FPI levels for the total study population (FPI-6 ≥ +6, *n* = 290; FPI-6 ≥ +8, *n* = 142; FPI-6 ≥ +10, *n* = 41). Given the expected variation in BMI/age group, the overweight children/mean FPI-6, and overweight children with flatfeet, were also calculated for each year of age and each FPI level (FPI-6 ≥ +6, *n* = 74; FPI-6 ≥ +8, *n* = 36; FPI-6 ≥ +10, *n* = 9) (Table 3).

**Table 2:** The study population according to age groups, and flatfeet

Age (years)	No. children	No. children with flatfeet/FPI-6 cut-off level		
		*+6*	*+8*	*+10*
3	21	15	11	1
4	23	14	8	1
5	40	14	6	3
6	37	15	6	2
7	31	13	9	3
8	176	56	7	1
9	63	26	13	6
10	110	40	26	12
11	126	54	32	3
12	80	35	20	7
13	13	5	3	2
14	6	2	1	0
15	1	1	0	0
Total no. children	728	290	142	41

It is evident that at the increasing FPI cut-off levels, that the numbers of children with flat feet diminished: FPI +6 - 290 (39.8 %); FPI +8 - 142 (19.5 %); FPI +10 – 41 (5.6 %)

**Table 3:** The study population according to age groups, BMI cut-offs, and overweight children

Age	No. children	BMI – cut off points/age (IOTF)		No. overweight children (mean FPI)	No. overweight children with flatfeet for each FPI-6 cut-off		
		*Male*	*Female*		*+6*	*+8*	*+10*
3	21	17.89	17.56	5 (7.0)	3	3	0
4	23	17.55	17.28	1 (3.0)	0	0	0
5	40	17.42	17.15	10 (2.6)	0	0	0
6	37	17.55	17.34	11 (5.7)	3	1	1
7	31	17.92	17.75	11 (5.7)	4	1	1
8	176	18.44	18.35	63 (3.6)	10	1	0
9	63	19.10	19.07	67 (3.9)	16	3	1
10	110	19.84	19.86	37 (4.8)	11	8	4
11	126	20.55	20.74	39 (4.9)	17	11	1
12	80	21.22	21.68	24 (4.3)	9	7	1
13	13	21.91	22.58	4 (4.0)	1	1	0
14	6	22.62	23.34	0	0	0	0
15	1	23.29	23.94	0	0	0	0
Total no. children	728	-	-	272	74	36	9

The overweight children with flatfeet tapered at each FPI cut-off level: 74 (10.1 %) at FPI ≥ +6, 36 (4.9 %) at FPI ≥ +8, and 9 (1.2 %) at FPI ≥ +10

## Discussion

In contrast to the previous studies cited in Table 1, which also investigated the relationship between body mass and foot posture in children, this study has repeated and confirmed our earlier finding [5], refuting the finding of flatter feet in heavier children.

This study comprised a broad age range of healthy children and foot types, which is important for the external validity of these findings.

Tables 2 and 3 indicate that at the FPI ≥ +6 level, approximately 40 % of the children were defined as having flatfeet and that almost 40 % of children were overweight. The percentage of flatfeet reduced to 20 % at FPI ≥ +8, and five percent at FPI ≥ +10.

In this study, ten percent of children who were overweight, in accordance to the IOTF cut-off levels [16], also had flatfeet when FPI scores ≥ +6 were applied. This reduced to five percent for FPI ≥ +8, and to one percent for FPI ≥ +10. This indicates that just 1:10 (10.1 %) children with flatfeet were also overweight with FPI ≥ +6, 1:20 (4.9 %) at FPI ≥ +8, 1:100 (1.2 %) at FPI ≥ +10, whilst greater concern was the finding of overweight in approximately 1:3 (37.3 %) of children.

This study is a secondary analysis of the acquired data, and is not without limitation. The reliability of the examiners in each of the component studies is not known, although the FPI-6 has well reported inter-rater reliability [12, 18], and is widely used in both clinical practice and research as a static measure of foot posture [17, 19]. Further limitation is possible given 67 subjects with Sever’s disease, which may result in heel pain related to physical activity, which could confound the relationship between BMI and foot posture. As an investigation subsequent and comparative to an earlier work [5], this investigation necessarily used the same basic parameters (i.e. FPI-6 > +6), and the IOTF cut-off levels for BMI/age. Given the wide age range of the 728 children in the complete dataset (three to 15 years), the FPI score cut-off at +6 may have been set too low for younger children, as the normative data suggest a range of +2 to +9 for children (mean score +3.7 (SD 2.5) – rounds to +4) [17]. The normative FPI data for children has been based on a sample of 397 children, aged from three to 17 years (average age 8.5 years) [17]. The data set for the present study included 728 children of similar age range (average age 9.07 years), with mean FPI-6 scores rounding to +5 (range −3 to +12). In accordance with the available normative data, the FPI cut-off set at +6 represents the normative mean FPI score + 1 SD, and hence captured those children with flatfeet which were ‘potentially abnormal’ [17], given the average age of nine years.

The higher FPI cut-off scores (i.e. FPI-6 > +8, and i.e. FPI-6 > +10), enable the existing normative data to be more broadly applied and to encompass those children with flatfoot posture that was more ‘probably abnormal’ (mean FPI score approximating: +4 + 2SD = +8) and those considered ‘pathological’ (mean FPI score approximating: +4 + > 2SD = +10) [17]. Of interest, is that the normative dataset also identified no relationship between FPI and BMI (*r* = 0.026, *p* = 0.574) [17].

It can be argued that the BMI is not an ideal measure for paediatric adiposity or body morphology [20, 21]. However, like the FPI, the BMI is widely used in both clinical practice and research, and was the available parameter common to all datasets to represent body mass. The IOTF cut-off levels for each year of age helps to specify and account for BMI variation across childhood [16], and were applied in this study’s analysis in order to appreciate the simultaneous and differing expected changes in both foot posture and body mass which occur with growth and development.

An important point that arises from this study, and indeed all of the studies that have investigated the relationship between body mass and children’s foot posture, is that the association between factors cannot be regarded as causal. Whilst the majority of studies investigating the relationship between foot posture and body mass have concluded that flatter feet are found in heavier children, this association must be cautioned, as indeed the findings of the current study indicate no association between children’s foot posture and body mass.

As was identified in our previous investigation, a principal difference between the findings of this study and all others investigating the relationship between children’s foot posture and body mass, was the method of classifying foot posture [5]. The FPI-6 is an observational scale that rates six aspects of foot anatomy, in contrast to the footprint based measures, which have been more widely used.

The issue central to the assessment of paediatric foot posture, by either a footprint based measure or the FPI-6, is the validity of either measure. It has been both suggested and refuted that footprint based measures may actually gauge adiposity, versus foot skeleton architecture [22, 23]. Foot print validity based on x-rays as the criterion standard has been most extensively evaluated by Villarroya et al. [24], who examined 58 obese children aged between nine and 16 years, matched with a control group of 58 normal weight children. Footprint based measures showed significantly flatter feet in the obese group versus the normal weight controls. However, this study was limited in that the radiographic measures were only applied to the obese group, with reference to normal values derived by Vanderwilde in only 12 nine year-old children [25]. Whilst the lateral talo-first metatarsal angle and calcaneo-inclination angle indicated flatfeet in the obese children, there was no comparison with the control group, which greatly limits the findings, as there was no established radiographic difference in foot posture between obese and normal weight children. Hence, the Villarroya study in attempting to validate the footprint based measures, did not clarify whether or not ‘flatter’ footprints in obese children were due to the weighted expanse of adipose tissue, nor whether or not the medial longitudinal arch x-ray angles differ in children who are obese versus normal weight controls [24].

Further, the FPI-6, whilst found to have good inter-rater reliability, construct validity [26], and validation against a Rasch statistical model [18], has not been validated against a criterion standard, and its use in children is less explored than in adult subjects.

As the current study has confirmed the previous finding [5] of there being no relationship between increased body mass and flatter feet in a large sample of children, the next area of inquiry is to explore the methods by which children’s foot posture is assessed. The difference between footprints, and foot posture as determined by the FPI-6, is now the focus of future projects.

Whilst there is little doubt about the need for concern about childhood overweight and obesity from a wider health perspective [27], clinicians need to be careful in currently interpreting any specific concern about flattening foot posture [28] in overweight children as this is not supported by the results of this study.

## Conclusions

This study supports our earlier findings, and conflicts with many other studies, in finding no relationship between increased BMI and ‘flatter’ feet in children. Whilst the inherent properties of the BMI and FPI are limiting, these findings question the concern about children’s BMI as a specific influence on (flatter) foot posture, and also the validity of footprint based versus anatomically based measures. Research now needs to explore the relationship between clinical measures of children’s foot posture and reference imaging.

## Consent

Written informed consent was obtained from the patient’s guardian/parent/next of kin for the publication of this report and any accompanying images.
